# Management and Diagnosis of Empagliflozin-Induced Burning Mouth Syndrome: A Case Report

**DOI:** 10.7759/cureus.70701

**Published:** 2024-10-02

**Authors:** Rui Seixas, Pedro Pessegueiro

**Affiliations:** 1 Stomatology Department, Hospital de São Bernardo, Unidade Local de Saúde da Arrábida, Setúbal, PRT; 2 Escola de Gestão, Engenharia e Aeronáutica, Instituto Superior de Educação e Ciências Lisboa, Lisboa, PRT; 3 Nephrology Department, Hospital do Espírito Santo de Évora, Unidade Local de Saúde do Alentejo Central, Évora, PRT

**Keywords:** burning mouth syndrome (bms), burning sensation in oral mucosa, empagliflozin, oral diseases, sglt2-inhibitors

## Abstract

Burning mouth syndrome (BMS) poses a significant clinical challenge. Patients often present with symptoms that can severely affect their quality of life, leading to anxiety, depression, and social withdrawal. The etiology of BMS remains poorly understood, which complicates its diagnosis and treatment. This case report describes an 80-year-old woman who presented with BMS following the administration of empagliflozin, a sodium-glucose cotransporter-2 (SGLT2) inhibitor used for glycemic control, along with its benefits in the progression and prognosis of cardiac insufficiency and chronic renal disease. Despite multiple treatments, her symptoms persisted until empagliflozin withdrawal, resulting in significant improvement at the six-month follow-up. This report suggests a potential association between empagliflozin and BMS, underscoring the need for clinicians to be vigilant regarding drug-related etiologies in the diagnosis and management of oral symptoms.

## Introduction

Burning mouth syndrome (BMS) is a clinical condition characterized by a persistent burning sensation in the oral mucosa without any identifiable clinical or laboratory abnormalities [1]. While additional symptoms such as dryness (xerostomia) and taste disturbances (dysgeusia) may accompany it, BMS can manifest solely as a burning sensation, reinforcing its recognition as an independent entity [2]. The exact underlying mechanisms remain unclear; however, the condition is believed to result from a multifactorial interplay involving neurological, hormonal, psychological, and local factors [3].

BMS affects approximately 0.7% to 15% of the population, predominantly among middle-aged and older women, particularly those who are postmenopausal [4]. Individuals with diabetes are at increased risk, possibly due to neuropathic changes associated with chronic hyperglycemia [5]. According to the International Classification of Headache Disorders, 3rd edition, BMS is defined as "an intraoral burning or dysesthetic sensation, recurring daily for more than two hours per day over more than three months, without clinically evident causative lesions" [2]. The burning sensation commonly affects the tongue (glossodynia), lips, and the anterior hard palate but can involve the entire oral cavity.

The etiology of BMS is multifactorial and can be classified into primary (idiopathic) and secondary forms [6]. In primary BMS, no underlying medical condition is identified, and neuropathic dysfunction is often suspected. Conversely, secondary BMS results from identifiable factors. Systemic conditions implicated include nutritional deficiencies-such as vitamin B12, folic acid, iron, and zinc deficiencies-endocrine disorders like hypothyroidism, hormonal imbalances including estrogen deficiency in postmenopausal women, and diabetes mellitus [1,3]. Local factors that may contribute to the condition include oral candidiasis, chronic mechanical irritation from dental appliances, and allergic reactions to dental material. Psychological factors, including anxiety, depression, and chronic stress, have also been associated with BMS, suggesting a psychogenic component in some patients [4].

Empagliflozin is a sodium-glucose co-transporter-2 (SGLT2) inhibitor indicated for the management of type 2 diabetes mellitus. It enhances glycemic control by inhibiting SGLT2 in the proximal renal tubules, thereby reducing glucose reabsorption and promoting urinary glucose excretion [7]. Beyond glycemic management, empagliflozin has demonstrated significant cardiovascular and renal benefits. The EMPA-REG OUTCOME trial revealed that empagliflozin significantly reduced the risk of major adverse cardiovascular events (MACE), cardiovascular death, and hospitalization for heart failure in patients with type 2 diabetes and established cardiovascular disease [8]. Additionally, empagliflozin has been shown to slow the progression of kidney disease, reduce albuminuria, and decrease the risk of renal endpoints, thus contributing to renal protection [9].

Drug-related oral health issues are significant because they can adversely affect a patient's quality of life, nutritional status, and medication adherence. Recognizing potential adverse effects on the oral cavity is essential for comprehensive patient care and early intervention.

## Case presentation

An 80-year-old woman, partially dependent on her daily activities, was referred to the stomatology department because of a persistent oral burning sensation that lasted for over four months. The patient described a moderate to severe burning sensation (visual analog scale (VAS) score 7-8/10) primarily localized to the anterior and middle tongue, both lips, superior and inferior gingivae as well as the buccal mucosa-especially in the anterior region. The sensation worsened as the day progressed, peaking in the early afternoon and remaining intense throughout the rest of the day. The intensity of the burning sensation was highest in the anterior region of the tongue, followed by the middle region of the tongue and both the upper and lower gingiva. The sensation was of lower intensity in both the upper and lower lips. The burning was not accompanied by dryness or any major changes in taste perception. The symptoms were daily, continuous, without specific triggers, and did not alleviate with changes in diet or oral hygiene practices.

The burning sensation impaired the patient’s ability to perform routine activities. Eating became difficult; she reported avoiding spicy and salty foods to prevent intensification of the oral burning. Although speaking was already affected by the lack of dental prosthesis, the patient experienced additional discomfort during speech. Upon oral examination, the patient was completely edentulous in both jaws with a well-hydrated oral mucosa. No lesions were observed in the oral cavity. There were no signs or previous history of oral dryness, candidiasis, dental trauma, mucosal erosions, ulcerations, or any changes in the oral mucosa.

The relevant medical history included chronic kidney disease and noninsulin-treated type 2 diabetes mellitus without complications such as diabetic retinopathy, nephropathy, or neuropathy. Her cardiovascular history included chronic arterial hypertension, atrial fibrillation, congestive heart failure with moderate tricuspid insufficiency, pulmonary hypertension, and biauricular dilation. Regular medications included linagliptin, zolpidem, valsartan, furosemide, edoxaban, bisoprolol, and allopurinol. Furthermore, she had started empagliflozin treatment four months prior, to improve her glycaemic control and its advantages for the progression and prognosis of cardiac insufficiency and chronic renal disease. The patient did not start any new medications or increase the dosages of current medications around the time of the initiation of empagliflozin.

Laboratory analysis revealed no significant abnormalities. For the therapeutic management of BMS, topical oral clonazepam (2.5 mg/ml) was prescribed three times daily for one month. The initial score for the visual analog scale (VAS) was 8/10. Following the absence of therapeutic effects, gabapentin (300 mg/day) was introduced for two months, without any improvement, and the VAS score remained at 7/10. These therapies were selected based on their documented effectiveness, safety profiles, particularly relevant in this case, and their specific action on neuropathic mechanisms underlying BMS. Given the possibility of adverse effects of empagliflozin, this medication was discontinued, which led to significant symptomatic improvement, with the VAS score decreasing to 2/10. A follow-up six months after discontinuation showed that the patient no longer experienced symptoms of oral burning, with the VAS score at 1/10 and 0/10 at the third and sixth month, respectively.

## Discussion

The management of multiple comorbidities in patients with type 2 diabetes mellitus can lead to polypharmacy, increasing the risk of adverse events. Managing polypharmacy in these patients requires a multifaceted approach to prevent drug-induced conditions like BMS. Clinicians should conduct regular medication reviews to identify potentially harmful drug interactions and unnecessary medications. This involves assessing the patient’s complete medication list, including over-the-counter drugs and supplements [1]. Deprescribing, the process of systematically discontinuing medications that are no longer needed or that pose more risks than benefits, is crucial [10]. Clinicians should prioritize medications based on their necessity and the patient’s overall health goals.

Monitoring for symptoms of BMS, such as a burning sensation in the mouth, is essential. If BMS is suspected, clinicians should evaluate the patient’s medications to identify potential culprits. Adjusting dosages or switching to alternative medications with a lower risk of causing BMS can be effective. Additionally, educating patients about the importance of medication adherence and potential side effects can empower them to report any adverse symptoms. Regular follow-ups and open communication between healthcare providers and patients are vital to ensure optimal medication management and to address any emerging issues promptly [1,6,10].

By implementing these strategies, clinicians can effectively manage polypharmacy in diabetes patients and reduce the risk of drug-induced conditions like BMS [10]. In the present case, the onset of BMS was linked to the initiation of empagliflozin treatment, as supported by the timing of symptom onset and resolution after discontinuation. Identifying the underlying etiology of BMS, which is mostly a diagnosis of exclusion, is essential for medical management [1,11]. The differential diagnosis of BMS requires consideration of local factors, such as oral candidiasis, ill-fitting oral prostheses, allergic reactions to physical or chemical compounds, and oral mucosal lesions, which can be easily ruled out through oral evaluation and patient history. Systemic factors, including nutritional deficiencies like vitamin B12, folate, iron, and zinc, or systemic diseases such as gastroesophageal reflux, thyroid disorders, and diabetes, can also be evaluated based on symptoms, medical history and changes in blood tests [1,12]. While diabetes mellitus can cause BMS, it did not correlate with the onset of symptoms or progression of the condition in this case, as demonstrated by the temporal relationship with empagliflozin. To the best of our knowledge, this is the first report of a BMS associated with empagliflozin. Many medications, including antihypertensives, antiretrovirals, and antidepressants, have been associated with BMS [12-14]. Notably, recent research has increasingly highlighted the potential side effects of newer drugs, including SGLT2 inhibitors such as empagliflozin, in various populations [7,14,15].

Although SGLT2 inhibitors are primarily designed for glycemic control in diabetes management and, more recently, for its cardiac and renal protection, they have been associated with various oral adverse effects, for example, xerostomia, which can contribute to or worsen BMS symptoms [7]. Despite the multifactorial nature of BMS, it's possible that medications like empagliflozin could potentially trigger or exacerbate oral burning symptoms in some individuals. While exploring different hypotheses on how empagliflozin induces BMS, we acknowledged the possibility of mechanisms that are still not fully understood. A proposed hypothesis by the authors is that empagliflozin could modulate the oral microbiome. By altering glucose levels in body fluids, for example in saliva and/or gingival crevicular fluid, empagliflozin may affect the oral microbiome's composition. Changes in the oral microbial balance can influence local inflammation and mucosal health. An imbalance in the oral microbiome might provoke inflammatory responses or increase mucosal sensitivity, contributing to the development of BMS [16].

A recent study has highlighted that drug-induced oral adverse effects, such as glossitis, tongue edema, and burning sensations, are relatively common among patients taking medications for chronic conditions [17]. These adverse effects can be attributed to the pharmacological action of the drugs, which may alter salivary flow, mucosal integrity, and oral microbial balance, leading to discomfort and pain in the oral cavity [18,19]. Investigating the connection between pharmacological therapies and emerging oral syndromes will allow clinicians to offer suitable treatment recommendations. As healthcare providers become more vigilant about medication side effects, awareness of the possible BMS symptoms linked to specific drugs is important in clinical practice, paving the way for early intervention and management strategies.

## Conclusions

Healthcare professionals must be aware of the potential role of certain medications in the pathogenesis of BMS. This awareness is becoming increasingly important, particularly for drugs like empagliflozin, which have not yet been widely associated with this condition. As the advantages of SGLT2 inhibitors for cardiovascular, renal, and metabolic health gain recognition, their use has seen a significant increase. However, this rise has also resulted in more adverse effects, with this report identifying BMS as one of them.

Therefore, it is imperative to manage this syndrome effectively to prevent the discontinuation of these medications and to maintain the benefits they offer. A proactive approach may lead to better outcomes for patients with BMS and enhance our understanding of the relationships between pharmacological treatment and oral health.
